# Novel approaches to the analysis of family data in genetic epidemiology

**DOI:** 10.3389/fgene.2015.00027

**Published:** 2015-02-06

**Authors:** Nathan Morris, Robert C. Elston, Jill S. Barnholtz-Sloan, Xiangqing Sun

**Affiliations:** ^1^Department of Epidemiology and Biostatistics, Case Western Reserve UniversityOH, USA; ^2^Center for Clinical Investigation, Case Western Reserve UniversityOH, USA; ^3^Case Comprehensive Cancer Center, Case Western Reserve University School of MedicineOH, USA

**Keywords:** genome-wide association, family studies, study designs, genetic factors, environmental factors

## The importance of family data

The study of Genetic Epidemiology has historically focused on the inheritance of genetic factors and phenotypes within families. In fact, much of genetics involves the study of patterns of familial resemblance and identifying the factors that explain the observed patterns. However, in recent years the most common study design for investigating the genetic determinants of diseases has become that of genome wide association studies (GWAS) utilizing samples of *unrelated* individuals. The popularity of this approach has been driven primarily by a flood of ever improving technologies. Unfortunately, while GWAS using unrelated individuals have revealed a great many interesting disease associated variants, these variants are typically of small effect and cannot explain the observed patterns of heritability for many traits. In contrast there are numerous examples of highly penetrant rare segregating alleles that have been discovered using family based approaches. Furthermore, family based approaches have other advantages: the ability to overcome confounding factors such as population stratification, and the numerous studies that have collected large amounts of family data and which should continue to be leveraged. Unfortunately, family based approaches to genetics have an added layer of complexity at all stages from design to analysis.

This editorial introduces the Frontiers in Genetics Research Topic and Ebook: “Novel approaches to the analysis of family data in genetic epidemiology.” The papers in this issue reveal that, even with easy access to high-throughput genotyping tools such as SNP arrays and next generation sequencing, family based study designs still play an important role in untangling the complex web of environmental and genetic factors that lead to disease.

## Family based study designs

A number of articles in this issue shed light on unique study designs and approaches to analyzing family data. Stein et al. (2013) describe a household contact study design which involves collecting data on households that may include both related and unrelated individuals. They argue that this research study design may be a powerful approach for jointly studying genetic and environmental exposures. Similarly, Estus et al. (2013) describe an approach to combining family based and population based data by utilizing a combined association test. Wang et al. (2013) describe an approach of using only the independent probands from a family based study of autism to investigate genetic factors that account for IQ differences in autism patients. Nelson et al. (2013) describe a unique population based registry in Utah that contains pedigree information for all residents of the state and dates back many decades. Using this information they show that certain subsets of prostate cancer, such as early onset, high BMI, and lethal prostate cancer, cluster in families more strongly than other forms of prostate cancer. They further suggest that future studies should focus on families that display a clear clustering of a more carefully defined cancer phenotype to reduce the signal to noise ratio. Uemoto et al. (2013) discuss the power of regional heritability mapping with a mixed model approach applicable to both related and unrelated persons. This approach leverages the fact that even distantly related individuals share small regions of the genome that are inherited from a common ancestor.

## Analysis of family data

The analysis of family data is generally more complex than the analysis of unrelated samples, and, thus, specialized statistical methods and software are often needed. Huang et al. (2013) propose a novel method of linkage analysis using sequence data on large pedigrees. This method, which uniquely combines MCMC based approximations with non-stochastic approaches, can be used to map disease genes using linkage and/or association evidence. Song and Elston (2013a) investigate the distributional properties of a commonly used linkage analysis statistic. These authors also describe a new web based software package which, among other things, plots pedigrees, calculates genetic similarity coefficients and performs visualization of the relatedness among family members (Song and Elston, 2013b). Similarly, Lutz et al. (2013) describe a method of using data from family based studies to test for a direct genetic effect, an extension of a method previously used for analysis of unrelated individuals. Additionally, Lutz et al. (2014) describe an approach to look at secondary phenotypes in case-control genetic association studies that circumvents the computational issues of a former approach.

## Conclusion

Although GWAS with unrelated samples have become one of the most common study designs currently used in human genetics, utilizing a family based design has many advantages. If a variant can be observed to co-segregate with a phenotype within a family, the evidence for its association with the disease is greatly strengthened. Family data provide excellent opportunities to find highly penetrant rare variants, and thus discover important biology informing us about disease. The articles in this issue illustrate how family based genetic designs remain a foundational part of human genetics.

### Conflict of interest statement

The authors declare that the research was conducted in the absence of any commercial or financial relationships that could be construed as a potential conflict of interest.
